# FSHD muscle shows perturbation in fibroadipogenic progenitor cells, mitochondrial function and alternative splicing independently of inflammation

**DOI:** 10.1093/hmg/ddad175

**Published:** 2023-10-19

**Authors:** Elise N Engquist, Anna Greco, Leo A B Joosten, Baziel G M van Engelen, Peter S Zammit, Christopher R S Banerji

**Affiliations:** Randall Centre for Cell and Molecular Biophysics, King's College London, New Hunt's House, Guy's Campus, London SE1 1UL, United Kingdom; Department of Neurology, Donders Institute for Brain, Cognition and Behaviour, Radboud University Medical Center, Nijmegen, 6525 GA, The Netherlands; Department of Internal Medicine, Radboud Institute of Molecular Life Sciences (RIMLS) and Radboud Center of Infectious Diseases (RCI), Radboud University Medical Center, Geert Grooteplein Zuid 10, Nijmegen 6525 GA, The Netherlands; Department of Internal Medicine, Radboud Institute of Molecular Life Sciences (RIMLS) and Radboud Center of Infectious Diseases (RCI), Radboud University Medical Center, Geert Grooteplein Zuid 10, Nijmegen 6525 GA, The Netherlands; Department of Medical Genetics, Iuliu Hatieganu University of Medicine and Pharmacy, 400012, Cluj-Napoca, Romania; Department of Neurology, Donders Institute for Brain, Cognition and Behaviour, Radboud University Medical Center, Nijmegen, 6525 GA, The Netherlands; Randall Centre for Cell and Molecular Biophysics, King's College London, New Hunt's House, Guy's Campus, London SE1 1UL, United Kingdom; Randall Centre for Cell and Molecular Biophysics, King's College London, New Hunt's House, Guy's Campus, London SE1 1UL, United Kingdom; The Alan Turing Institute, The British Library, 96 Euston Road, London NW1 2DB, United Kingdom

**Keywords:** facioscapulohumeral muscular dystrophy (FSHD), fibroadipogenic progenitor cells (FAPs), transcriptomics, mitochondrial function, alternative splicing

## Abstract

Facioscapulohumeral muscular dystrophy (FSHD) is a prevalent, incurable myopathy. FSHD is highly heterogeneous, with patients following a variety of clinical trajectories, complicating clinical trials. Skeletal muscle in FSHD undergoes fibrosis and fatty replacement that can be accelerated by inflammation, adding to heterogeneity. Well controlled molecular studies are thus essential to both categorize FSHD patients into distinct subtypes and understand pathomechanisms. Here, we further analyzed RNA-sequencing data from 24 FSHD patients, each of whom donated a biopsy from both a non-inflamed (TIRM^−^) and inflamed (TIRM^+^) muscle, and 15 FSHD patients who donated peripheral blood mononucleated cells (PBMCs), alongside non-affected control individuals. Differential gene expression analysis identified suppression of mitochondrial biogenesis and up-regulation of fibroadipogenic progenitor (FAP) gene expression in FSHD muscle, which was particularly marked on inflamed samples. PBMCs demonstrated suppression of antigen presentation in FSHD. Gene expression deconvolution revealed FAP expansion as a consistent feature of FSHD muscle, via meta-analysis of 7 independent transcriptomic datasets. Clustering of muscle biopsies separated patients in an unbiased manner into clinically mild and severe subtypes, independently of known disease modifiers (age, sex, D4Z4 repeat length). Lastly, the first genome-wide analysis of alternative splicing in FSHD muscle revealed perturbation of autophagy, BMP2 and HMGB1 signalling. Overall, our findings reveal molecular subtypes of FSHD with clinical relevance and identify novel pathomechanisms for this highly heterogeneous condition.

## Introduction

Facioscapulohumeral muscular dystrophy (FSHD) is an autosomal dominant myopathy with a prevalence of ~12/100 000 [1] that typically presents during the second or third decade of life. FSHD is characterized by muscle weakness and a progressive loss of muscle mass that typically begins in the facial muscles before progressing to the shoulder girdle and lower limb [2–4]. However, a high degree of inter-patient variability is observed in age of onset, muscle groups affected and rate of progression. FSHD also associates with extra-muscular features including sensorineural, high-frequency hearing loss and retinal telangiectasia, with increased prevalence in more severely affected patients [5–7].

FSHD comprises two genetically distinct subtypes: FSHD1 (OMIM: 158900, ~95% of cases) and FSHD2 (OMIM: 158901, ~5% of cases). Both subtypes result in epigenetic derepression of the D4Z4 macrosatellite region in the telomeric region of chromosome 4q35 [8]. This is due to contraction of the D4Z4 region from the healthy range of 100-11 D4Z4 repeats to 10-1 on at least one allele in FSHD1 [9, 10], or mutations in chromatin modifiers in FSHD2 [11, 12]. Either event enables transcription of the *Double homeobox 4 (DUX4)* (OMIM: 606009) retrogene from the distal-most D4Z4 unit [13–15]. When combined in *cis* with a permissive 4qA haplotype supplying a poly(A) signal, the stabilized ectopic *DUX4* mRNA [11] can be translated to generate DUX4 protein, which is believed to be the key driver of FSHD pathogenesis [7, 16, 17].

DUX4 is a pioneer transcription factor involved in zygotic genome activation during the cleavage stage [18]*,* which is then epigenetically silenced in somatic tissues. Re-expression of DUX4 and its target genes in FSHD is linked to myotoxicity both *in vitro* [3, 19–26] and *in vivo* [27–31]. Mechanisms of DUX4 myotoxicity include mitochondrial dysfunction [21, 32–34], altered muscle regeneration through functional interference with PAX7 [26, 35, 36], increased exposure and susceptibility to oxidative stress [22, 37–40], p53-dependent apoptosis [24, 25] and alterations in RNA splicing [41–43]. However, full understanding of the mechanisms through which DUX4 drives pathology in FSHD remains elusive.

Although DUX4 is very difficult to detect/undetectable in patient biopsies using routine lab methods [15], signatures based on DUX4 target gene expression, or PAX7 target gene repression, associate with pathological severity and disease duration [26, 35, 36]. Further pathomechanisms identified from discovery-based analysis of FSHD muscle biopsy transcriptomics include Wnt/β-catenin signalling [44, 45], HIF1α overactivation [26, 44], TNFα overactivation [21, 44], mis-regulation of vascular genes [46], perturbed myogenesis [47], altered inflammatory signalling [48, 49] and RAGE-NF-κb signalling [39].

FSHD muscle is characterized by inflammation, which accelerates underlying fatty replacement of muscle and drives clinical weakness [7, 50]. As such, histological and transcriptomic analyses of skeletal muscle of FSHD patients are often accompanied by magnetic resonance imaging (MRI) to select inflamed muscles, which are identifiable by oedema using inversion recovery MRI sequences (e.g. TIRM, STIR) [51–55]. Such studies demonstrate that numbers of regenerating fibres in FSHD muscle positively correlate with severity [54] and inform several transcriptomic signatures of FSHD that can distinguish FSHD muscle from control with varying degrees of accuracy [26, 36, 49, 55–57].

Myogenic cells are not the only perturbed cell type in FSHD, implicating non-myogenic cell types resident in muscle, particularly fibroadipogenic progenitor (FAP) cells [53, 58]. FAPs comprise a heterogenous mesenchymal cell population, which interact with immune cells and muscle stem cells to facilitate muscle regeneration. Mis-regulation of FAPs contributes to fibrosis and fat infiltration in other forms of muscular dystrophy [59]. Recent work indicates a similar mis-regulation of FAPs may exist in FSHD [53, 58]. Peripheral blood mononuclear cells (PBMCs) from FSHD patients have been examined to develop minimally invasive blood-based biomarkers of disease progression. These studies suggest a potential role for IL-6 and identify transcriptomic signatures shared across FSHD muscle and blood [48, 60–62].

In addition to genetics, there are many sources of heterogeneity in FSHD muscle, with factors such as inflammation driving intra-patient variability, and external factors such as lifestyle, contributing to inter-patient variability. As such, clinical heterogeneity in FSHD patients must be controlled when evaluating tissue-specific effects such as inflammation. Well-designed transcriptomic studies of multiple FSHD tissues are essential to better understand molecular features of FSHD, facilitate biomarker discovery and guide therapeutic development.

Here, we report further transcriptomic analysis of isogenic MRI-guided biopsies of both non-inflamed (TIRM^−^) and actively inflamed (TIRM^+^) muscles and PBMCs from the same FSHD patients, alongside muscle-matched biopsies and PBMCs from healthy controls [60]. Our analyses indicate FAP population expansion and mitochondrial dysfunction in FSHD muscle, which progress with inflammation. Our transcript-level investigation provides global analysis of RNA splicing in FSHD muscle, detecting differences in post-transcriptional regulation with pathological relevance that were previously masked by gene-level aggregation. Finally, we describe potential defects in antigen presentation in FSHD PBMCs.

## Results

### Differential gene expression of FSHD muscle biopsies reveals transcriptomic profiles associated with clinical severity

We further considered our data corresponding to 49 individuals: 25 FSHD1 patients, 1 FSHD2 patient and 23 control individuals, described in detail in [60]. Briefly, clinical variables obtained included: age, sex and MRC sum score for 12 muscle groups. For FSHD patients, severity indicators measured included: D4Z4 repeat length (for FSHD1 patients), Ricci clinical severity score [63], Lamperti clinical severity score [64], disease duration, lower limb fat fraction (LLFF) assessed by MRI and maximum voluntary contraction (MVC) of tibialis anterior (TA) [60].

As we described previously [60], muscle biopsies were obtained from 35/49 individuals for RNA-sequencing. 23 FSHD1 patients and 1 FSHD2 patient underwent MRI guided muscle biopsies with samples taken from TIRM^−^ (23/24 vastus lateralis) and TIRM^+^ muscle (15/24 gastrocnemius) from each patient. 11 control individuals also donated a muscle biopsy of vastus lateralis. FSHD patients who underwent muscle biopsy were significantly older than controls and showed a male sex bias [60].

**Figure 1:**
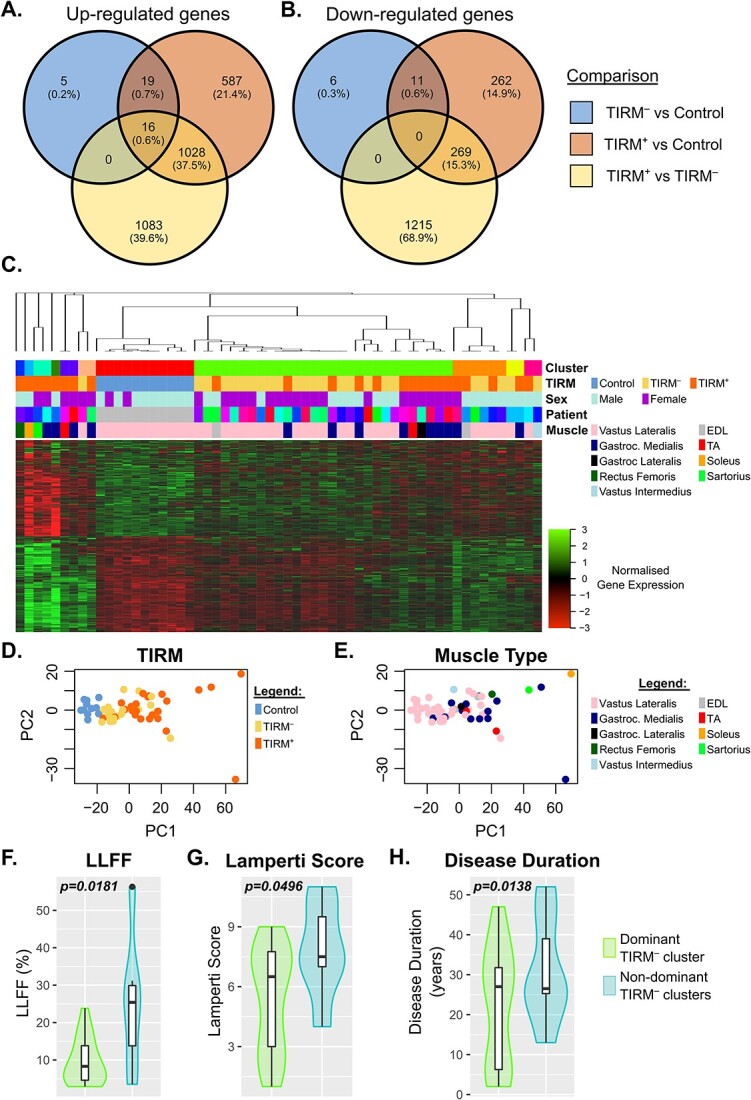
Differential gene expression analysis of muscle biopsies reveals clinically relevant FSHD subtypes. (A, B) Venn diagrams display overlaps in genes significantly (A) up-regulated and (B) down-regulated in comparisons between non-inflamed (TIRM^−^) FSHD muscle versus control (blue), inflamed (TIRM^+^) FSHD muscle versus control (orange) and TIRM^+^ vs TIRM^−^ FSHD muscle (yellow). (C) Heatmap displays the optimal 11 cluster solution of 56 muscle biopsy samples, employing 1015 genes significantly altered in TIRM^−^/TIRM^+^ FSHD muscle versus control as a feature space. *z*-normalized gene expression is coloured red to green and samples are labelled with TIRM and FSHD status, sex, muscle and patient identifier. (D, E) Scatter plots display samples according to the first and second principal components derived from the same 1015 gene feature space used for clustering. Samples are labelled with (D) TIRM status and (E) muscle type. (F–H) Boxplots display values of (F) LLFF, (G) Lamperti score and (H) disease duration for the 18 TIRM^−^ FSHD muscle biopsies which were assigned to the dominant FSHD cluster (green) compared to the 6 which lay outside this cluster (blue). Multivariate regression *P*-values assessing association with each clinical variable and dominant cluster status are displayed, adjusting for age, sex and muscle type.

Two separate differential gene expression analyses were performed on the muscle biopsy data, analysing length adjusted transcript counts aggregated to gene level via negative binomial models using the *DESeq2* package [65]. First, we performed a multivariate analysis to identify genes whose expression differed across control, TIRM^−^ and TIRM^+^ samples, adjusting for age and sex. Comparing TIRM^−^ FSHD samples to controls independently of TIRM^+^, we identified 17 genes down-regulated and 40 genes up-regulated after adjustment for multiple testing. Comparing TIRM^+^ FSHD samples to control independent of TIRM^−^, we identified 1650 genes up-regulated and 542 down-regulated after adjustment for multiple testing (Fig. 1A and B, Supplementary Table S1). This suggests that TIRM^−^ samples are more subtly changed from control muscle in terms of the gene expression landscape compared to TIRM^+^ samples.

Second, we focused on FSHD samples and performed a paired analysis comparing gene expression in isogenic TIRM^−^ and TIRM^+^ samples from each FSHD patient. Directly comparing isogenic TIRM^−^ and TIRM^+^ FSHD samples, we identified 2127 genes up-regulated in TIRM^+^ samples compared to matched TIRM^−^ samples and 1484 genes down-regulated. There were overlaps in genes identified in the two analyses, with 16 genes consistently up-regulated in TIRM^−^ versus control, TIRM^+^ versus control and TIRM^+^ versus TIRM^−^, but no genes consistently down-regulated in the three comparisons (Fig. 1A and B).

*DUX4* was only detected in 2 TIRM^−^ and 4 non-corresponding TIRM^+^ FSHD samples, and undetectable in both the remaining 42/48 FSHD samples and all (11/11) control samples. Where detected, DUX4 was at very low levels (max 3 reads per sample) and was not differentially expressed between FSHD and control samples, nor between matched TIRM^−^ and TIRM^+^ samples.

We next performed a clustering analysis of FSHD and control samples. As a feature space, we used all genes differentially expressed in TIRM^−^ samples versus controls and the 500 most significantly up-regulated or down-regulated genes in TIRM^+^ samples versus controls, as assessed by adjusted *P*-value (1015 genes in total). K-medoids clustering using a Pearson correlation distance metric was performed using the *ConsensusClusterPlus* algorithm [66] and cluster stability plots indicated an 11 cluster solution (Fig. 1C). Principal component analysis (PCA) demonstrated that the dominant principal component was associated with TIRM^−^ and TIRM^+^ status, independently of age, sex and muscle type (multivariate co-efficient *P*-value TIRM^−^ = 0.0049, TIRM^+^ = 0.00722, Fig. 1D). The dominant muscle types (vastus lateralis and gastrocnemius medialis) were not associated with the dominant principal component. However, the single soleus and sartorius TIRM^+^ samples did separate from the remaining muscle samples by the dominant principal component (multivariate co-efficient *P*-value soleus = 0.0007, sartorius = 0.025, Fig. 1E). There was no association between the dominant principal component and FSHD patient matched pair samples, indicating that inter-individual variability does not drive major variability in expression of genes in our feature space.

Importantly, one of the 11 clusters solely contained only control muscle biopsy samples, indicating that genes comprising the feature space are indeed perturbed in FSHD. Of the remaining 10 clusters containing only FSHD samples, the dominant cluster comprised 29 samples of which 62% (18 samples) are from the 24 TIRM^−^ samples and 38% (11 samples) are from the 24 TIRM^+^ samples. The remaining clusters contained either 6, 2 or 1 samples and are made up by the majority (54%, 13/24 samples) of TIRM^+^ samples and the 6 TIRM^−^ samples.

TIRM positivity indicates an inflammatory state associated with faster disease progression [50]. Given the imperfect separation of TIRM^−^ and TIRM^+^ samples, we postulated that TIRM^+^ samples in the dominant, majority TIRM^−^ cluster have milder clinical severity compared to FSHD samples outside this cluster. To address this, we first considered TIRM^−^ samples, 18 of which lay in the dominant cluster and 6 outside. Multivariate regression was performed to determine association between clinical severity and membership of the dominant cluster, using a variety of clinical assessments: Ricci clinical severity score, Lamperti clinical severity score, MRC sum score, LLFF, disease duration, MVC of TA and D4Z4 repeat length, adjusting for age, sex and muscle (where possible). The 18 TIRM^−^ samples in the dominant cluster displayed significantly lower LLFF (multivariate regression *P*-value = 0.0181, Fig. 1F) and Lamperti clinical severity scores (multivariate regression *P*-value = 0.0496, Fig. 1G) together with significantly shorter disease duration (multivariate regression *P*-value = 0.0138, Fig. 1H), compared to 6 TIRM^−^ samples outside this cluster, independently of patient age, sex and muscle. This is consistent with TIRM^−^ samples outside the dominant cluster being more clinically affected.

We next considered TIRM^+^ samples, 11 of which lay in the dominant cluster and 13 outside. There was no association between cluster membership and clinical variables for TIRM^+^ samples, after adjustment for age, sex and muscle.

### Fibroadipogenic progenitor cell gene expression hallmarks FSHD muscle and discriminates TIRM^+^ from TIRM^−^ status

**Figure 2:**
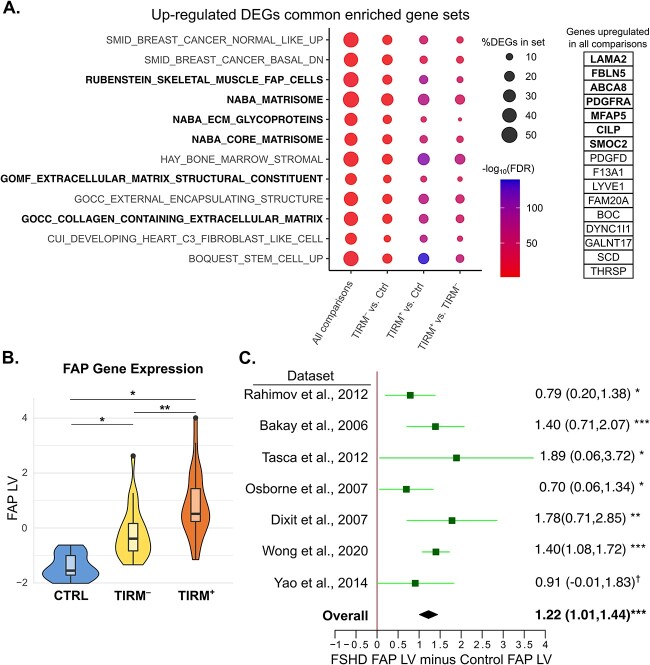
Fibroadipogenic progenitor (FAP) gene expression is up-regulated in FSHD muscle biopsies and increases with inflammation. (A) Dot plot heatmap displays gene sets in MSigDB which were consistently enriched among differentially expressed genes from four comparisons: The 16 genes up-regulated in all comparisons (listed beside the plot), all genes up-regulated in TIRM^−^ versus control, the 500 most significant genes up-regulated in TIRM^+^ versus control, the 500 most significant genes up-regulated in TIRM^+^ versus TIRM^−^ FSHD muscle. The size of the dot is proportional to the percentage of up-regulated genes found in each enriched gene set, dots are coloured red to blue according to −log_10_(FDR). The FAP gene set is highlighted in bold text, as are the 7/16 genes up-regulated in all comparisons which overlap with the FAP gene set. Gene sets related to ECM maintenance are also in bold text. (B) Boxplot displays the value of the latent variable associated with FAPs and no other muscle biopsy cell type (FAP LV) following deconvolution of our 56 muscle biopsy samples employing the approach of Rubenstein *et al*. [67]. Samples are separated into control (blue), TIRM^−^ (yellow) and TIRM^+^ (orange). An asterisk denotes a significance of *P* < 0.05 and two asterisks denotes *P* < 0.01 from multivariate regression determining association of the FAP LV with control vs TIRM^−^ or TIRM^+^ status independently of age and sex, or a paired comparison of matched TIRM^−^ and TIRM^+^ samples. (C) Forrest plot displays the difference between FAP LV values in FSHD and control samples, following deconvolution of 7 independent FSHD muscle biopsy microarray or RNA-seq datasets (130 FSHD, 98 control). In 6/7 datasets and on meta-analysis, the FAP LV values are significantly elevated on FSHD samples, implying increased FAP gene expression in FSHD. Boxes denote the mean difference in FAP LV between FSHD and control muscle biopsies and whiskers denote 95% confidence interval. A vertical line denotes a FAP LV difference of 0 and datasets where the whiskers cross this line have not attained significance at *P* < 0.05 (as assessed by Wilcoxon test). Values for mean FAP LV difference and confidence interval are displayed for each dataset with significance level, where an asterisks denotes *P* < 0.05, two asterisks denotes *P* < 0.01, three asterisks denotes *P* < 0.001, and † denotes *P* = 0.052). The overall estimate is displayed as a diamond and was computed using a random effects model with significance assessed via Fisher’s combined test.

We next investigated the function of the genes differentially expressed in our FSHD samples via gene set enrichment analyses (GSEA). We found the FAP gene set defined by Rubenstein *et al*. [67] to be the most enriched gene set in the 40 genes up-regulated in TIRM^−^ FSHD muscle versus control (adjusted *P*-value = 1.3 × 10^−5^  Fig. 2A), and highly enriched in the 500 genes most significantly up-regulated when comparing TIRM^+^ muscle to control (adjusted *P*-value = 4.2 × 10^−88^  Fig. 2A) and TIRM^+^ muscle to isogenic TIRM^−^ muscle samples (adjusted *P*-value = 6.8 × 10^−44^, Fig. 2A). This same gene set was also the most highly enriched among the 16 genes up-regulated in all three differential gene expression analyses and among these genes is the well characterized FAP marker *PDGFRA* (Fig. 2A). Additionally, several gene sets pertaining to FAP cell function in ECM deposition and maintenance were strongly enriched across comparisons (Fig. 2A).

Given this clear identification of gene expression signatures for FAPs in FSHD, we next assessed the relative contribution of FAPs to the gene expression landscape of each muscle biopsy using the deconvolution algorithm described by Rubenstein *et al*. [67] with the *PLIER* package in R [68]. As implied by the GSEA results, we found that both TIRM^−^ and TIRM^+^ FSHD muscle biopsies had significantly higher FAP gene expression compared to control muscle biopsies independently of age, sex and muscle (multivariate *P*-value TIRM^−^ = 0.027, TIRM^+^ = 0.026, Fig. 2B). Moreover, isogenic TIRM^+^ biopsies had significantly higher FAP gene expression compared to TIRM^−^ biopsies from the same patient (*P* = 0.0018, Fig. 2B).

Elevated FAP gene expression in FSHD muscle has also been found in a previous study of largely non-isogenic muscle biopsies [55]. To investigate how consistent this finding is we analyzed a further 7 independent transcriptomic datasets profiling FSHD and control muscle biopsies, employing the same *PLIER* algorithm to determine FAP contribution in each sample. Strikingly, 6/7 datasets demonstrated significant up-regulation of FAP gene expression in FSHD samples compared to controls, and the seventh approached significance (*P* = 0.052) (Fig. 2C). This up-regulation was highly significant on meta-analysis (Fisher’s combined *P* = 1.4 × 10^−16^, Fig. 2C).

### Genes associated with mitochondrial function and metabolism are suppressed in FSHD muscle

**Figure 3:**
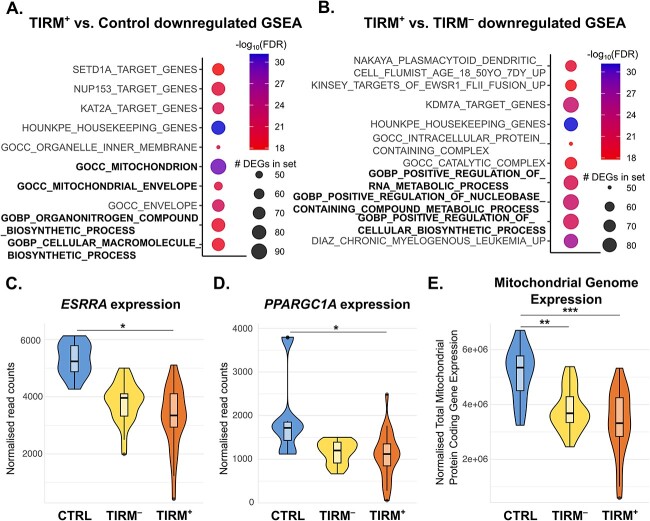
Mitochondrial gene expression and key biogenesis regulators are suppressed in FSHD muscle. (A, B) Dot plot heatmaps displays gene sets in MSigDB which were most significantly enriched among (A) the 500 most significant genes down-regulated in TIRM^+^ versus control and (B) the 500 most significant genes down-regulated in TIRM^+^ versus TIRM^−^ FSHD muscle. The size of the dot is proportional to the number of down-regulated genes in each enriched gene set, dots are coloured red to blue according to −log_10_(FDR). Mitochondrial and metabolic gene sets are in bold text. (C, D) Boxplots display normalized read counts for (C) *ESRRA* and (D) *PPARGC1A* across control (blue), TIRM^−^ (yellow) and TIRM^+^ (orange) samples, where an asterisk denotes adjusted *P* < 0.05 from DESeq analysis of differential expression, adjusting for age, sex and matched pair. (E) Boxplot of the sum of normalized read counts for the 13 protein coding mitochondrial genes across control, TIRM^−^ and TIRM^+^ samples. Two asterisks denotes *P* < 0.01, and three asterisks *P* < 0.001 from multivariate regression determining the association of mitochondrial genome expression with control vs TIRM^−^ or TIRM^+^ status independently of age and sex.

We next investigated genes suppressed in FSHD muscle via GSEA. There were no significantly enriched gene sets among the 17 genes suppressed in TIRM^−^ biopsies versus controls. GSEA on the 500 most down-regulated genes in TIRM^+^ biopsies versus controls demonstrated clear enrichment for mitochondrial genes and nucleic acid metabolism (Fig. 3A). GSEA on the 500 most down-regulated genes in the paired comparison of TIRM^−^ to TIRM^+^ biopsies also revealed strong enrichment for metabolic processes (Fig. 3B).

We have previously demonstrated insufficient mitochondrial biogenesis in differentiating human myoblasts from FSHD patients, which is linked to inadequate activation of ERRα by PGC1α [69]. Given suppression of mitochondrial genes and metabolic processes in our new FSHD patient biopsies, we specifically examined *ESRRA*, encoding ERRα, and *PPARGC1A*, encoding PGC1α. Expression of both *ESRRA* and *PPARGC1A* was suppressed in TIRM^+^ muscle compared to control (Fig. 3C and D), confirming our in vitro findings [69] in FSHD patients.

Additionally, total expression of all 13 protein-coding genes in the mitochondrial genome, a metric shown to correlate with mitochondrial DNA copy number in skeletal muscle [70] and several types of cancer [71], was significantly suppressed in both TIRM^−^ and TIRM^+^ samples compared to controls independently of age and sex. This indicates lower mitochondrial content in FSHD samples (Fig. 3E).

### Differential transcript analysis of FSHD muscle biopsies demonstrates perturbation of autophagy and PAX7 target genes

To examine alternative splicing, we next performed differential transcript analyses using *DRIMSeq* [72] and *stageR* [73] packages in R. Adjusting for matched pairs imposed strict restrictions on the transcript distribution across samples, limiting the number of genes we could assess in a manner which may introduce bias. We therefore focused on comparisons of control versus TIRM^−^ or TIRM^+^ status, adjusting for age and sex of participants. We followed an established protocol to identify genes which displayed evidence of both differential transcript usage (DTU) and differential transcript expression (DTE) [74].

**Figure 4:**
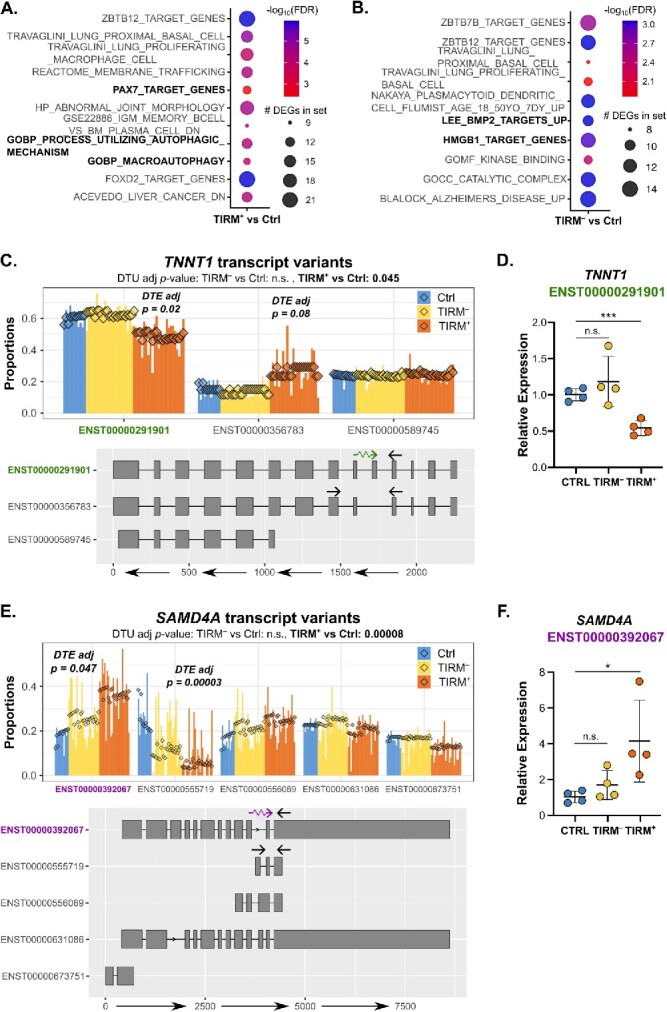
Alterative splicing analysis in muscle biopsies reveals novel FSHD changes. (A, B) Dot plot heatmaps displays gene sets in MSigDB which were most significantly enriched among genes alternatively spliced in (A) TIRM^+^ versus control and (B) TIRM^−^ versus control. The size of the dot is proportional to the number of alternatively spliced genes found in each enriched gene set, dots are coloured red to blue according to −log_10_(FDR). PAX7 target genes, autophagic processes and BMP2 and HMGB1 target genes in bold text. (C–F) Bar plots generated using our bespoke software (Supplementary File S1) display the proportion of total gene expression for (C) *TNNT1* and (E) *SAMD4A* accounted for by the major transcript variants, across 11 control samples (blue), 24 TIRM^−^ FSHD samples (yellow) and 24 TIRM^+^ FSHD samples (orange). Transcript structures are displayed beneath each plot and coloured text highlights and explains the transcript that is significantly altered in its expression in TIRM^−^ (yellow) or TIRM^+^ (orange). At the top of each plot adjusted *P*-values are displayed denoting the significance of differential transcript usage (DTU) for the gene between control and TIRM^−^ and control and TIRM^+^ samples (adjusted for age, sex and multiple comparisons). On each plot is displayed the adjusted *P*-value for differential transcript expression (DTE) of the highlighted transcript in the comparison for which DTU is significant, adjusting for age, sex and multiple comparisons. Validation of (D) *TNNT1* and (F) *SAMD4A* expression of highlighted isoforms was performed by quantitative real-time PCR in 4 control samples (blue), 4 TIRM^−^ samples (yellow) and their paired isogenic TIRM^+^ samples (orange), using RNA from samples selected on predicted DTE and quantity of RNA available. *TNNT1* ENST00000291901 expression was quantified relative to sum expression of ENST00000291901 and ENST00000356783, and *SAMD4A* ENST00000555719 expression was quantified relative to that of ENST00000392067, the other isoform with DTE. Primer locations are indicated by arrows on the transcript structures in (C) and (D). Data is presented as ΔΔCt relative to controls, with each point representing the mean of 3 technical replicates per biopsy. Black bars represent group mean ± SD, and significance is indicated by an asterisk that denotes *P* < 0.05 or 3 asterisks that denote *P* < 0.01.

We identified 64 genes with evidence of DTU and DTE in TIRM^−^ samples versus controls and 121 genes in TIRM^+^ samples versus controls, with an overlap of 25 genes in both comparisons (Supplementary Table S2). GSEA on the 121 genes alternatively spliced in TIRM^+^ muscle versus control revealed strong enrichment for autophagic processes, immune processes and PAX7 target genes, an interesting finding considering PAX7 target gene are repressed in FSHD muscle [26, 35, 36] (Fig. 4A). The 64 genes alternatively spliced in TIRM^−^ muscle versus control were enriched for a number of processes including target genes of both BMP2, a signalling protein that inhibits adipogenic differentiation of FAP cells [75], and the nuclear DNA-binding protein HMGB1, which modulates muscle inflammation [76] and regulates PAX7 expression [77] during myogenesis (Fig. 4B).

Among genes alternatively spliced in TIRM^+^ muscle versus control is *TNNT1*, the slow muscle troponin, which has two main isoforms showing signs of differential usage: ENST00000356783 (TNNT1-202), which lacks 11 amino acids encoded by exon 5 of the higher molecular weight ENST00000291901 (TNNT1-201). We see specific down-regulation of the high molecular weight TNNT1-201 in TIRM^+^ FSHD muscle compared to control, with a trend to up-regulation of the low molecular weight TNNT1-202 (Fig. 4C). We designed primers to distinguish these two isoforms and confirmed down-regulation of the high molecular weight TNNT1-201 in TIRM^+^ FSHD muscle compared to control by RT-qPCR in a subset of our muscle biopsy samples (Fig. 4D).

A second example of an alternatively spliced gene is *SAMD4A*, encoding the RNA-binding translational repressor SMAG1 protein, which exhibits compensatory DTE in two transcript isoforms between control and TIRM^+^ samples. TIRM^+^ samples up-regulate ENST00000392067, which encodes the canonical protein, while control samples up-regulate ENST00000555719 (SAMD4A-209), a severely truncated isoform consisting of 3 exons (Fig. 4E). Again, we designed primers to distinguish these two isoforms and confirmed up-regulation of the transcript encoding canonical SMAG1 in TIRM^+^ FSHD muscle compared to control by RT-qPCR in a subset of our muscle samples (Fig. 4F).

Among genes exhibiting differential splicing patterns in TIRM^−^ muscle versus control was *MYH14*, a non-muscle myosin heavy chain, with 2 main spliced isoforms: ENST00000376970 (NMHCII-C0) which lacks 8 amino acids within the globular head domain encoded by exon 6, compared to ENST0000042560 (NMHCII-C1). We found up-regulation of NMHCII-C0 specifically in FSHD TIRM^−^ muscle compared to control (Supplementary Fig. S1A). A second gene exhibiting DTU in TIRM^−^ muscle compared to controls is *RFX5*, a transcriptional activator of major histocompatibility complex (MHC) class II promoters. Compared to controls, TIRM^−^ samples exhibit up-regulation of ENST00000412774 (RFX5-204), a severely truncated isoform lacking sequences encoding most of the N-terminus and part of the C-terminus (Supplementary Fig. S1B).

To facilitate independent investigation of the splicing patterns of other genes in TIRM^−^ and TIRM^+^ FSHD muscle compared to control, we have developed a data visualization tool, written in *shiny* R [78]. The programme displays the transcript structure, relative expression in control, TIRM^−^ and TIRM^+^ muscle biopsies and corresponding *P*-values for any given gene with significant DTU in our above comparisons (Supplementary File S1).

### Differential gene expression of FSHD and control peripheral blood mononuclear cells (PBMCs) demonstrates suppression of antigen processing and presentation

RNA-sequencing had also been performed on peripheral blood mononuclear cells (PBMCs) that were isolated from 14 FSHD1 patients, 1 FSHD2 patient and 14 control patients [60]. We have previously demonstrated no difference in age or sex distribution between control and FSHD PBMC samples in this cohort [60]. Differential expression analysis was performed analysing length adjusted transcript counts aggregated to gene level, via negative binomial models using the *DESeq2* package [65], to determine gene expression associated with FSHD PBMCs, adjusting for age and sex.

**Figure 5:**
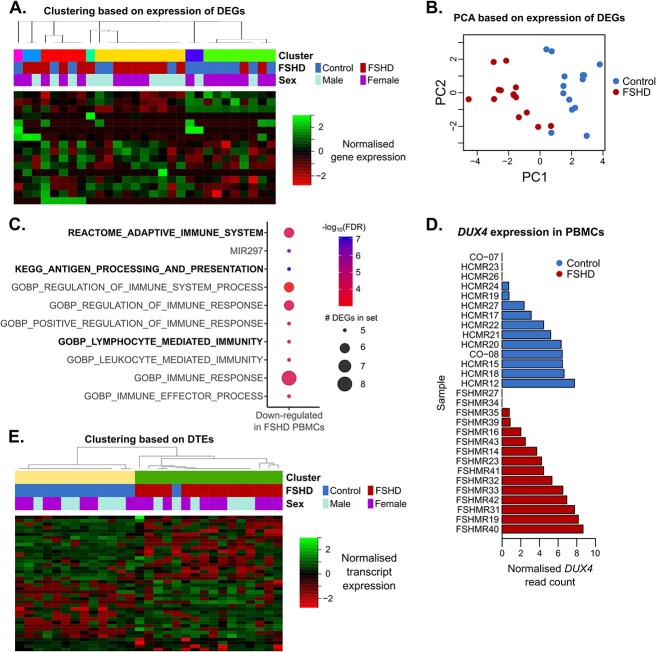
FSHD PBMCs display suppression of antigen presentation and consistent alternative splicing features. (A) Heatmap displays the optimal 7 cluster solution of 29 PBMC samples, employing 16 genes significantly altered in FSHD vs control PBMCs, expressed in >3 samples as a feature space. *z*-normalized gene expression is coloured red to green and samples are labelled with FSHD status and sex. (B) Scatter plot display samples according to the first and second principal components derived from the same 16 gene feature space used for clustering, labelled with FSHD status. (C) Dot plot heatmap displays gene sets in MSigDB which were most significantly enriched among genes suppressed in FSHD PBMCs. The size of the dot is proportional to the number of suppressed genes found in each enriched gene set, dots are coloured red to blue according to −log_10_(FDR). Adaptive immunity gene sets are highlighted in bold text. (D) Bar plot displays normalized read count for *DUX4* in each control (blue) and FSHD (red) PBMC sample. (E) Heatmap displays the optimal 2 cluster solution of 29 PBMC samples, employing 40 transcripts with evidence for DTE within genes with evidence for DTU in FSHD vs control PBMCs as a feature space. *z*-normalized transcript expression is coloured red to green and samples are labelled with FSHD status and sex.

Gene expression differences between FSHD and control PBMCs were more subtle than muscle and we identified only 26 genes up-regulated and 21 genes down-regulated in FSHD PBMCs compared to controls after adjusting for multiple comparisons. Of these 47 genes, only 16 were expressed in more than 3 samples. Using expression of these 16 genes as a feature space, K-medoids clustering of the 29 PBMC samples revealed a 7 cluster solution with poor separation of FSHD and control samples (Fig. 5A), despite the dominant principal component associating with FSHD status, independently of age and sex (multivariate regression *P* = 9.7 × 10^−9^_,_  Fig. 5B).

GSEA on the 26 genes up-regulated in FSHD PBMCs demonstrated no enriched gene sets. However, GSEA on the 21 genes down-regulated in FSHD PBMCs demonstrated enrichment for antigen processing and presentation as well as adaptive immunity (Fig. 5C).

As per muscle, DUX4 was again not differentially expressed between FSHD and control samples. However, in contrast to muscle biopsies, DUX4 transcripts were expressed in 13/15 FSHD patient and 11/14 control PBMC samples. Expression of DUX4 in PBMCs was low but higher than seen in muscle biopsies (max 8 reads per sample in FSHD and 6 in controls, Fig. 5D).

### Differential transcript expression but not differential gene expression permits near perfect separation of FSHD and control PBMCs

We next performed DTU and DTE analysis to identify alternative splicing events in FSHD PBMCs compared to controls, adjusting for age and sex of participants. We identified 36 genes with evidence of DTU and DTE in FSHD PBMCs. GSEA on these genes revealed no significantly enriched gene sets.

The 36 genes yielded 40 transcripts with evidence of differential expression between FSHD and control samples. Given that differentially expressed genes facilitated poor separation of FSHD and control PBMCs via clustering analysis, we repeated clustering analysis using expression of these 40 transcripts as a feature space. K-medoids clustering yielded a 2 cluster solution which almost perfectly separated FSHD and control samples, with the exception of a single control sample (Fig. 5E). This indicates that the FSHD state is better discriminated in PBMCs by consideration of differential transcript profiles than differential gene expression.

As with the muscle biopsies, to facilitate independent investigation of splicing patterns in PBMCs we have developed a data visualization in the *shiny* package [78] in R (Supplementary File S2).

## Discussion

We have investigated differential gene expression, differential transcript usage and differential transcript expression in isogenic non-inflamed (TIRM^−^) and inflamed (TIRM^+^) FSHD muscle biopsies alongside matched control muscle biopsies, as well as in FSHD and control PBMCs.

FSHD is highly heterogeneous, with monozygotic twins often progressing at dramatically different rates [79]. This unpredictable heterogeneity is a key stumbling block to FSHD clinical trials, where for example, unintentional overloading of treatment arms with fast progressors could mask detection of treatment efficacy. In similarly heterogenous pathologies such as breast cancer, molecular subtyping is extremely useful in identifying homogenous subgroups of patients, facilitating personalized medicine approaches [80, 81]. D4Z4 repeat length has been linked with accelerated progression in FSHD [82] as has male sex [83], and MRI studies show that inflammation can accelerate clinically relevant fatty replacement in FSHD muscle [50]. We have also demonstrated that FSHD patients can follow a number of distinct clinical phenotypes, independently of sex and D4Z4 repeat length [84]. However, clinically relevant molecular subtypes that provide the basis of a testable, quantitative grouping have not been described in FSHD.

Here we show that expression of 1015 genes, differentially expressed in TIRM^−^ and TIRM^+^ FSHD muscle compared to controls, separates our samples into a control cluster and 10 distinct FSHD clusters. The largest FSHD cluster contains TIRM^−^ muscle samples which are clinically milder than TIRM^−^ muscle samples in the other FSHD clusters, with lower Lamperti clinical severity scores, lower LLFF and shorter disease duration, despite equal D4Z4 repeat length and independent of age and sex. This is the first evidence that clinically relevant molecular subtypes of FSHD exist, motivating investigation of molecular subtype biomarkers in larger cohorts.

FAPs are interstitial muscle-resident multipotent progenitor cells, with capacity to differentiate into myofibroblasts, adipocytes and, under certain conditions, chondrocytes and osteocytes [75]. FAPs are activated and proliferate after muscle injury and differentiate following specific molecular cues into specialized cells that prepare the extra-cellular matrix to support muscle regeneration. Mis-regulation of FAP function has been implicated in aberrant muscle regeneration in several neuromuscular disorders including LGMD2B [85], ALS [86] and recently FSHD [53, 55, 58]. FAP dysregulation in FSHD was first noted in a mouse model of stochastic transient DUX4 expression, which resulted in an increased FAP gene expression signature [58] that was validated histologically and found to associate with muscle fibrosis post injury [87]. Subsequently, an increased FAP transcriptomic signature was found in a dataset of 39 mainly non-isogenic FSHD muscle biopsies, where it correlated with FSHD transcriptomic biomarkers of severity [55]. Recently a detailed characterization of FSHD patient mesenchymal cells demonstrated increased numbers of CD201^+^ and PDGFRA^+^ FAPs in FSHD, which correlated with accelerated fibrosis and disease progression and showed inhibited adipogenesis ex vivo [53]. We found that FAP gene expression is the most enriched up-regulated gene set in FSHD muscle versus control. We analyzed isogenic samples and demonstrated that within patients, inflamed TIRM^+^ muscle displayed elevated levels of FAP gene expression to paired TIRM^−^ biopsies. Importantly, the FAP marker *PDGFRA* is up-regulated in TIRM^−^ and TIRM^+^ FSHD muscle versus control, as well as between matched TIRM^+^ and TIRM^−^ FSHD muscle. We confirmed this finding by performing meta-analysis of multiple independent FSHD published transcriptomic datasets, demonstrating that elevated FAP gene expression is a clear feature of FSHD muscle across 130 FSHD samples.

Mitochondrial dysfunction is also a well-studied feature of FSHD muscle [21, 33, 34, 69]. We have previously demonstrated that FSHD patient-derived myoblasts exhibit a regeneration defect characterized by small, hypotrophic myotubes [34, 69]. This is driven by suppression of mitochondrial biogenesis consequential of ERRα/PGC1α down-regulation [69], and dysfunctional mitochondria with elevated mitochondrial membrane potential and increased mitochondrial ROS generation [34]. ERRα agonist food supplements such as biochanin A, genistein and daidzein [69], as well as mitochondrially targeted anti-oxidants [34], can rescue such defective in vitro FSHD myogenesis. Here we show that mitochondrial genes are significantly suppressed in inflamed TIRM^+^ FSHD muscle, providing a direct patient level correlate of our in vitro finding. In particular genes encoding ERRα and PGC1α are significantly down-regulated in TIRM^+^ muscle, motivating translation of ERRα targeted food supplements to the clinic [69]. Reduced mitochondrial genome expression in both TIRM^−^ and TIRM^+^ FSHD samples further implies a progressive suppression of mitochondrial content in FSHD muscle.

Increased FAP-related gene expression and reduced mitochondrial gene expression in TIRM^−^ FSHD biopsies compared to muscle-matched controls indicates a baseline dysregulation of these processes in FSHD muscle regardless of inflammation.

The TIRM^−^ and control muscle biopsies were from the quadriceps femoris (with 34/35 being from vastus lateralis). However, since inflammation in FSHD muscle is sporadic and highly heterogenous between patients, it is rare to identify patients from which contralateral TIRM^−^ and TIRM^+^ samples can be taken from the same muscle. As such, the isogenic TIRM^+^ muscle samples come from a range of muscle types (although 15/24 were from gastrocnemius), raising the concern that gene expression changes in TIRM^+^ samples are due to transcriptional differences between different muscles [88]. While we cannot completely adjust for this covariate to eliminate the possibility of a muscle type contribution, principal component analysis on the genes used to identify expression patterns linked to TIRM status (such as FAPs and mitochondrial biogenesis) demonstrated strong association of the dominant principal component with TIRM status independently of muscle type (Fig. 1D and E). This indicates that differences in gene expression are more likely associated with TIRM status than muscle type in FSHD. Furthermore, several studies investigating human lower limb muscle transcriptomes have shown that variability in gene expression between individuals is substantially greater than that between muscle types within an individual [88, 89]. Thus for phenotypes such as inflammation, the noise contribution will be lower when comparing different muscle types within an individual than comparing the same muscle type across multiple individuals. Interestingly, such studies have also reported that certain muscles in healthy individuals, including those more commonly affected in FSHD (e.g. gastrocnemius), exhibit increased FAP gene expression and mitochondrial down-regulation compared to muscles such as the vastus lateralis [88]. This suggests that certain muscle groups may be predisposed to being more susceptible to pathological changes driving FSHD, which may explain the highly specific, but poorly understood, FSHD-affected muscle distribution.

TIRM^−^ FSHD muscle also down-regulated genes associated with RNA metabolism and nucleobase metabolism, a feature previously observed in DUX4 over-expressing myotubes, where it correlated with inhibited nonsense-mediated decay [90] and aberrant RNA splicing [41]. Thus, we performed the first genome wide analysis of differential transcript usage and expression in FSHD muscle. Genes with evidence of differential transcript expression in inflamed FSHD muscle were enriched for autophagic processes, of significance given an elevated level of autophagic vacuoles found in a case series of atypical FSHD patients [91], and a potential role for DUX4 in autophagy [92]. Curiously, enrichment for alternative splicing of PAX7 target genes in inflamed FSHD muscle biopsies was also observed. We have demonstrated, and others have confirmed, that PAX7 target genes are significantly suppressed in FSHD muscle [26, 55, 56], correlating with disease severity and progression [35, 36, 60]. It is possible that alternative splicing of PAX7 target genes drives exon suppression which in turn leads to PAX7 target suppression at the gene level, however dedicated studies would be necessary to test this hypothesis. Genes with differential transcript expression in TIRM^−^ FSHD muscle compared to control demonstrated enrichment of BMP2 and HMGB1 signalling. BMP2 drives osteogenic differentiation of FAPs while inhibiting adipogenic differentiation [75]. FAPs isolated from FSHD patients show inhibited adipogenic differentiation [53], while DUX4 expression is associated with osteogenesis [93]. HMGB1 is a critical mediator of muscle regeneration and inflammation in multiple muscular dystrophies [76]. Importantly, fully reduced HMGB1 contributes to muscle regeneration, while oxidized HMGB1 (e.g. by ROS) is a pro-inflammatory cytokine. HMGB1 also shares high structural homology with closely related family member HMGB2 [94], which is part of an inhibitory DNA binding complex that binds to the D4Z4 region at the DUX4 promoter [95]. Given that FSHD is a pathology characterized by elevated intracellular ROS [34], inhibited regeneration [54] and inflammation [50], further investigation of HMGB1 in FSHD is ongoing.

Several post-transcriptional misregulation events that we observed in FSHD muscle have precedent in pathologically relevant contexts. The precise splicing pattern of *MYH14* observed in TIRM^−^ samples compared to controls is also observed in Myotonic Dystrophy type 1 (DM1), a repeat expansion myopathy with associated sensorineural deafness [96]. *MYH14* is mutated in an autosomal dominant sensorineural deafness [97] and plays a role in mitochondrial fission [98], and both sensorineural deafness [6] and mitochondrial dysfunction [34, 69] are features of FSHD. The alternative splicing pattern of *TNNT1* observed in TIRM^+^ samples compared to controls resembles a pattern reported in muscle from patients with Charcot-Marie-Tooth disease type 1 (CMT1), but not CMT type 2, where it associates with increased specific force in individually isolated muscle fibres, as well as chronically overused muscle from a prior Polio patient [99]. The same splicing pattern of *TNNT1* has also been shown in muscles of trained older adults compared to sedentary participants, and again associated with increased fibre force independent of cross-sectional area [100].

Loss of *SAMD4A* has been linked to changes in RNA-binding and post-transcriptional regulation that can exacerbate myopathy [101] and cause metabolic defects such as uncoupling of mitochondrial respiration [102]. Increase in canonical *SAMD4A* in TIRM^+^ samples, rather than the truncated and likely non-functional isoform up-regulated in control samples, may reflect counteractive measures against metabolic defects affecting FSHD muscle. Finally, RFX5 is an essential member of the RFX complex, which binds to the X-box sequence in the promoter of MHC-II genes and facilitates transcriptional activation of MHC-II genes through association with other regulatory factors such as NF-Y [103, 104]. The *RFX5* isoform over-expressed in TIRM^−^ samples lacks part of the DNA-binding domain as well as a C-terminal sequence necessary for cooperative binding, both of which are necessary for activation of MHC-II gene expression [104]. This is interesting given the enrichment of adaptive immunity pathways in genes differentially expressed between control and FSHD PBMCs, particularly considering a report connecting *DUX4* expression in tumours to suppression of both MHC-I and MHC-II related gene expression [105, 106].

Together, these splicing patterns indicate that FSHD muscle may be adapting to a chronic pathogenic environment to improve muscle contractility, and that these adaptations are occurring in inflammatory stages of disease pathology prior to onset of fat infiltration. Deeper investigation of the remaining 183 alternatively spliced genes (Supplementary File S1) is ongoing to identify additional alternative splicing events with clinical relevance. While markers identified here in terms of gene expression and alternative splicing are limited to earlier, inflamed stages of muscle pathology, similar analyses in patient datasets containing a subset of muscles with fat infiltration [52, 55, 56] will inform whether these patterns observed persist through disease progression and may identify further transcriptional and/or post-translational perturbations affecting later stages of pathology.

Analysis of FSHD and control PBMCs demonstrated few differentially expressed genes, however genes suppressed in FSHD PBMCs were enriched for antigen presentation and processing. This is interesting given that DUX4 can suppress MHC class I expression in several malignancies in a manner associated with tumour immunotherapy resistance [106]. Suppression of antigen processing in FSHD PBMCs may therefore be reflective of a suppressed MHC class I presentation on DUX4 expressing cells throughout the patient such as skeletal muscle, making this an area of interest for future work. Curiously, DUX4 expression was higher in PBMCs than muscle biopsies, and both control and FSHD PBMCs expressed DUX4 transcripts. This is in line with the detection of DUX4 in immortalized lymphoblastoid cell lines from both FSHD and control individuals [48, 107]. Lastly, FSHD PBMCs showed evidence of differential transcript usage and expression compared to control PBMCs, and clustering based on differential transcript level, led to a more accurate separation of FSHD and control PBMCs compared to differential gene expression.

For ready investigation of the splicing patterns of other genes in TIRM^−^ and TIRM^+^ FSHD muscle and FSHD PBMCs compared to control, we provide data visualization tools (Supplementary Files S1 and S2). The programmes display the transcript structure, relative expression in control, TIRM^−^ and TIRM^+^ muscle biopsies and FSHD PBMCs and corresponding *P*-values for any given gene with significant DTU.

In summary, we performed a detailed transcriptomic analysis of isogenic FSHD non-inflamed and inflamed skeletal muscle as well as FSHD PBMCs. Our analyses indicate FAP cell population expansion and mitochondrial dysfunction in FSHD muscle, which progress in the presence of inflammation. Our transcript-level investigation provides global analysis of RNA splicing in FSHD muscle, detecting differences in post-transcriptional regulation with potential pathological relevance including BMP2 and HMBG1, which were previously masked by gene-level aggregation. Finally, we describe defects in antigen presentation in FSHD PBMCs indicating a potential role for DUX4 in systemic MHC I expression dynamics and propose transcript-level analysis as a powerful approach to blood based biomarker discovery.

## Materials and Methods

### Patient cohort

We have described the patient cohort before [60] but provide details here for reference. We considered data corresponding to 49 individuals: 26 FSHD patients (n = 25 FSHD1 and 1 FSHD2) and 23 unrelated unaffected control individuals, all investigated between 2019–2021 at the Radboud University Medical Center (Nijmegen, The Netherlands) Neurology Outpatient clinic. Subjects provided written informed consent and the study received regional medical ethical committee approval (CMO Arnhem-Nijmegen). Clinical data collected included: age of onset, FSHD type, D4Z4 repeat length, age of onset, disease duration, and LLFF assessed by MRI. Patients underwent muscle power assessment via the MRC sum score [108], in which muscle power grade (rated 0–5 per muscle) was assessed for 6 muscle groups on left and right, giving a sum score out of 60. FSHD patients also underwent the three additional clinical assessments: 1) the Ricci score [63], which ranges from 0–10 (0 = no symptoms, 10 = wheelchair bound) and assumes a typical progression of muscle weakness descending from face to legs, 2) the Lamperti score [64], a 0–15 severity scale (0 = low, 15 = high) which independently evaluates weakness of 6 muscle regions affected in FSHD to better account for atypical cases, and 3) MVC of tibialis anterior [109] when able. We have previously analyzed clinical variables and age and sex distributions in this dataset, showing Ricci, Lamperti and MRC sum scores are elevated in older, male FSHD patients [60]. FSHD patients who underwent muscle biopsy were significantly older than controls and showed a male sex bias [60]. A full overview of clinical data collected is provided in our previous publication [60].

### MRI acquisition

MRI imaging used to select muscle biopsy site was provided in detail previously [60] using a protocol established by Mul *et al*. [110]. To reiterate, Dixon sequences were analyzed using MATLAB and ImageJ software. MATLAB was used to calculate a fat fraction (FF) map from the water and fat image of the Dixon sequence according to: $FF=\frac{F}{F+W}$. Contours of twelve upper leg muscles (sartorius, gracilis, vastus medialis, vastus lateralis, vastus intermedius, rectus femoris, biceps femoris caput brevis, biceps femoris caput longus, semitendinosus, semimembranosus, adductor magnus, adductor longus) and seven lower leg muscles (tibialis anterior, extensor digitorum longus, peroneus, tibialis posterior, soleus, gastrocnemius medialis, and gastrocnemius lateralis) were outlined manually on the FF map as described [110], and average FF within a single muscle contour ($\overline{FF_i}$) was calculated in ImageJ. Average Lower limb fat fraction (LLFF) was calculated as follows: $LLFF=\frac{\sum_{i=1}^n{A}_i\cdotp \overline{FF_i}}{\sum_{i=1}^n{A}_i},$ where A = area of each muscle and n = muscle contours of one patient.

### Biopsy collection

As previously detailed [60], muscle biopsies had been collected from 24 FSHD individuals (23 FSHD1 and 1 FSHD2 patient) and 11 controls for RNA-sequencing. From each FSHD patient, two paired MRI-guided [111] muscle biopsies had been collected: one targeting a TIRM^−^ (non-inflamed) muscle (23/24 vastus lateralis, 1/24 vastus intermedius), and one from a muscle with presumed active inflammation selected based on the presence of TIRM hyperintensity, degree of fatty infiltration, and amount and location of normal appearing muscle (15/24 gastrocnemius). Bergström needle biopsies were collected from the vastus lateralis of all 11 control individuals as previously described [112].

### RNA-sequencing

RNA extraction, purification, quality control, library preparation, and sequencing was described previously [60]. Salient points are for PBMCs, globin depletion was performed prior to RNA extraction. Sequencing depth was 21.7–35.5 million reads/sample for muscle samples, and 19.7–46.5 million reads/sample for PBMCs. Raw reads were trimmed with TrimGalore (http://www.bioinformatics.babraham.ac.uk/projects/trim_galore/), using CutAdapt [113] to remove Illumina adapters at the 3′ end. An additional 15 bases were trimmed from the 5′ end of reads due to biased per base sequence content. Human genome sequence GRCh38 and v103 gene annotations were downloaded from Ensembl. Reads were mapped to the human transcriptome using *Salmon* [114] (v1.6.0), making use of the GC bias correction flag. The RNA-sequencing data from the isogenic FSHD muscle biopsies and PBMCs, and controls is described in Banerji *et al*. [60] and available upon reasonable request from The European Genome-phenome Archive (https://ega-archive.org) under accession number EGAS00001007350.

### Differential gene expression analysis and clustering

The *DESeq2* package [65] in R was used to aggregate length-adjusted transcript counts to the gene-level and perform differential gene expression analyses. A multivariate analysis, adjusting for age and sex, was used to identify differentially expressed genes (DEGs) between control vs. TIRM^−^ muscle, control vs. TIRM^+^ muscle, and control vs. FSHD PBMCs, adjusting for multiple comparisons. To identify genes whose expression differed between TIRM^−^ vs. TIRM^+^ samples, a paired analysis was performed between matched samples from individual patients, adjusting for multiple comparisons. Genes with adjusted *P*-value<0.05 in each analysis were considered significant.

K-medoids clustering was performed using the *ConsensusClusterPlus* algorithm [66] employing a Pearson correlation distance metric. For muscle biopsies, the clustering feature space comprised all genes differentially expressed in TIRM^−^ vs control samples and the 500 most significant (as assessed by adjusted *P*-value) genes up- and down-regulated in TIRM^+^ versus control samples, resulting in 1015 genes. For PBMCs, the feature space comprised all genes differentially expressed between control and FSHD PBMCs which were detectable in more than 3 samples, a feature space of 16 genes. The optimal number of clusters was assessed via consensus CDF plots.

Clinical variables (Ricci score, Lamperti score, MRC sum score, LLFF, disease duration, MVC of TA and D4Z4 repeat length) associated with TIRM^−^ FSHD samples assigned to the largest FSHD cluster were compared to those associated with TIRM^−^ FSHD samples assigned to other clusters via multivariate regression, adjusting for age, sex and muscle type. All but one of the TIRM^−^ FSHD samples are vastus lateralis, with the remaining sample vastus intermedius. MVC of TA could not be assessed in the single vastus intermedius sample due to weakness, and thus muscle type was not adjusted for in the regression performed against MVC of TA.

Principal component analysis was performed across muscle biopsy samples and PBMCs, employing the same feature space as for clustering. To confirm the main component of variability in our feature space associated with FSHD/TIRM status, multivariate regression analysis was performed to determine association between the dominant principal component and FSHD/TIRM status, adjusting for age and sex.

GSEA [115] was performed using Fisher’s Exact Test assessing overlap with gene sets compiled by the Molecular Signatures Database (MSigDB) [115]. The following gene sets were investigated: all genes up/down-regulated in TIRM^−^ vs control, the 500 most significant genes up/down-regulated in TIRM^+^ vs control, the 500 most significant genes up/down-regulated in TIRM^−^ vs TIRM^+^, the 16 genes up-regulated across all 3 comparisons (TIRM^−^ vs control, TIRM^+^ vs control and TIRM^+^ vs TIRM^−^), and all genes up/down-regulated in FSHD PBMCs vs controls, separately.

Deconvolution was performed as described by Rubenstein *et al*. [67] using the *PLIER* package [68] in R to derive latent variables associated with cell types identified in the single cell analysis of human muscle biopsies performed by Rubenstein *et al*. [67]. A single latent variable associated with FAPs but not with any other skeletal muscle cell type, and was assigned to represent the proportion of FAPs across the samples. Multivariate regression analysis was performed to assess association between the FAP associated latent variable and control vs TIRM^−^/TIRM^+^ status adjusting for age and sex. A separate multivariate regression was performed to compare matched TIRM^−^ and TIRM^+^ FSHD samples, adjusting for matched pairs. Significance was assessed at the 5% level.

Expression values of the 13 protein coding mitochondrial genes were summed over for each sample and compared as above via multivariate regression analyses.

### Meta-analysis of FAP contributions to FSHD muscle biopsies

Normalized transcriptomic data corresponding to FSHD and control muscle biopsies in seven independent studies [14, 46, 49, 51, 56, 116, 117] was obtained from the GEO database [118]. Rahimov *et al*. [117] GSE36398 describes 50 muscle biopsies assessed by microarray. Bakay *et al*. [116] GSE3307 describes 30 muscle biopsies assessed by microarray. Tasca *et al*. [51] GSE26852 describes 15 muscle biopsies assessed by microarray. Osborne *et al*. [46] GSE10760 describes 49 muscle biopsies assessed by microarray. Dixit *et al*. [14] GSE9397, describes 18 muscle biopsies assessed by microarray. Yao *et al*. [49] GSE56787 describes 23 muscle biopsies assessed by RNA-seq. Wong *et al*. [56] GSE115650 describes 43 muscle biopsies assessed by RNA-seq. Together, these seven datasets describe 228 muscle biopsies (130 FSHD, 98 control).

Each dataset was analyzed independently employing the deconvolution analysis outlined by Rubenstein *et al*. 2020 [67], using the *PLIER* package [68] in R. For each dataset, a latent variable was identified which associated with FAP cells but not with any other muscle cell type. A two-tailed Wilcoxon test was performed to assess the difference in FAP latent variable values assigned to FSHD vs control samples, with significance assessed at the 5% level. Meta-analysis across the seven independent studies were performed using a random effects model, and overall significance assessed via Fisher’s combined test.

### Transcript-level analyses

Differential transcript analyses were performed using *DRIMSeq* [72] and *stageR* [73] packages in R, using an established protocol [74]. *Salmon* transcript counts were imported to R using the scaled TPM option in the *tximport* package [119], a filter was applied to retain transcripts with a read count of at least 10 in all samples and with a relative abundance proportion of less than 0.1 in at least 10 samples. After filtering, *DRIMSeq* was employed to fit Dirichlet multinomial models to transcripts, identifying transcript distributions which differed between control, TIRM^−^ and TIRM^+^ muscle or between FSHD and control PBMCs, adjusting for age and sex and multiple testing. We identified genes with evidence of differential transcript usage (DTU) as those with adjusted *P*-values < 0.05. *DRIMSeq* also evaluated negative binomial models of differential transcript expression (DTE) between control, TIRM^−^ and TIRM^+^ muscle or between FSHD and control PBMCs, within genes with evidence of DTU, adjusting for age, sex and multiple hypotheses. Transcripts with adjusted *P*-values < 0.05 show evidence of DTE. Because DTE results from *DRIMSeq* have been shown to display an elevated overall false discovery rate (FDR) [74], we employed the *stageR* [73] package in R to identify significant transcripts for which to the overall FDR < 0.05. This refined set of transcripts were considered to show DTE.

GSEA was performed as above, investigating the genes sets containing genes with evidence for both DTU and DTE in TIRM^−^ vs control muscle, TIRM^+^ vs control muscle and FSHD vs control PBMCs.

K-medoids clustering was performed on the PBMC samples as above using the *ConsensusClusterPlus* algorithm [66] employing a Pearson correlation distance metric with a feature space comprising the expression of 40 transcripts with evidence of DTU and DTE between FSHD and control PBMCs.

A data visualization tool for genes which demonstrated evidence of DTU and DTE across our control, TIRM^−^ and TIRM^+^ muscle biopsies and control and FSHD PBMCs was developed using *shiny* R [78]. We used the *ggtranscript* package [120] to visualize transcript structure and the *DRIMSeq* package [72] to visualize DTU and DTE.

Codes written for analysis and visualization of data are available from the corresponding authors upon reasonable request.

### Real-time quantitative PCR

RNA was isolated from muscle samples by GeneWiz (https://www.genewiz.com/en-GB/), and reverse transcribed using QuantiTect Reverse Transcription kit (Qiagen 205313) as per manufacturer’s instructions. RT-qPCR was carried out using Takyon Low ROX SYBR 2X MasterMix blue dTTP (Takyon) as per the manufacturer’s instructions on a ViiA7 thermal cycler (Applied Biosystems). *TNNT1* ENST00000291901 (containing exon 5) expression was normalized to a region shared between ENST00000291901 and ENST00000356783 (the other isoform showing signs of differential usage), and *SAMD4A* ENST00000392067 (encoding full-length protein) was normalized relative to ENST00000555719 (truncated isoform). Expression values are presented as ΔΔCt relative to control. Primer sequences used were:

#### SAMD4A

ENST00000392067 Forward: 5′-CAGCAGCCAGAATTCCAGC-3′ ENST00000555719 Forward: 5′-CAGACCTTAGAGAATTCCAGCTTCC-3′ Reverse: 5′-GCGGTCACTCTCGTCTTCAG-3′

#### TNNT1

ENST00000291901 Forward: 5′-AGGAGGAAGCCCCCGAAG-3′ ENST00000291901/ENST00000356783 Forward: 5′-GAAGAGGAGGCTGCGGAG-3′ Reverse: 5′-CTTCTGGGATCTTTGGCGGG-3′

## Supplementary Material

Engquist_et_al_2023_Supplementary_Figure_1_ddad175

Engquist_et_al_2023_Supplementary_Table_1_ddad175

Engquist_et_al_2023_Supplementary_Table_2_ddad175

Engquist_et_al_ddad175_2023_Instructions_for_using_Supplementary_Files_S1_and_S2_ddad175

DTE_DTU_shiny_files_ddad175

DTE_DTU_shiny_files_PBMCs_ddad175

shiny_DTE_DTU_ddad175

shiny_DTE_DTU_PBMC_ddad175
