# Supplementary material for: FSHD muscle shows perturbation in fibroadipogenic progenitor cells, mitochondrial function and alternative splicing independently of inflammation
Source: Hum Mol Genet. 2023 Oct 19;33(2):182–97. doi: 10.1093/hmg/ddad175 (PMC10772042; doi:10.1093/hmg/ddad175)
Supplement: Engquist_et_al_2023_Supplementary_Figure_1_ddad175 [file engquist_et_al_2023_supplementary_figure_1_ddad175.pdf]

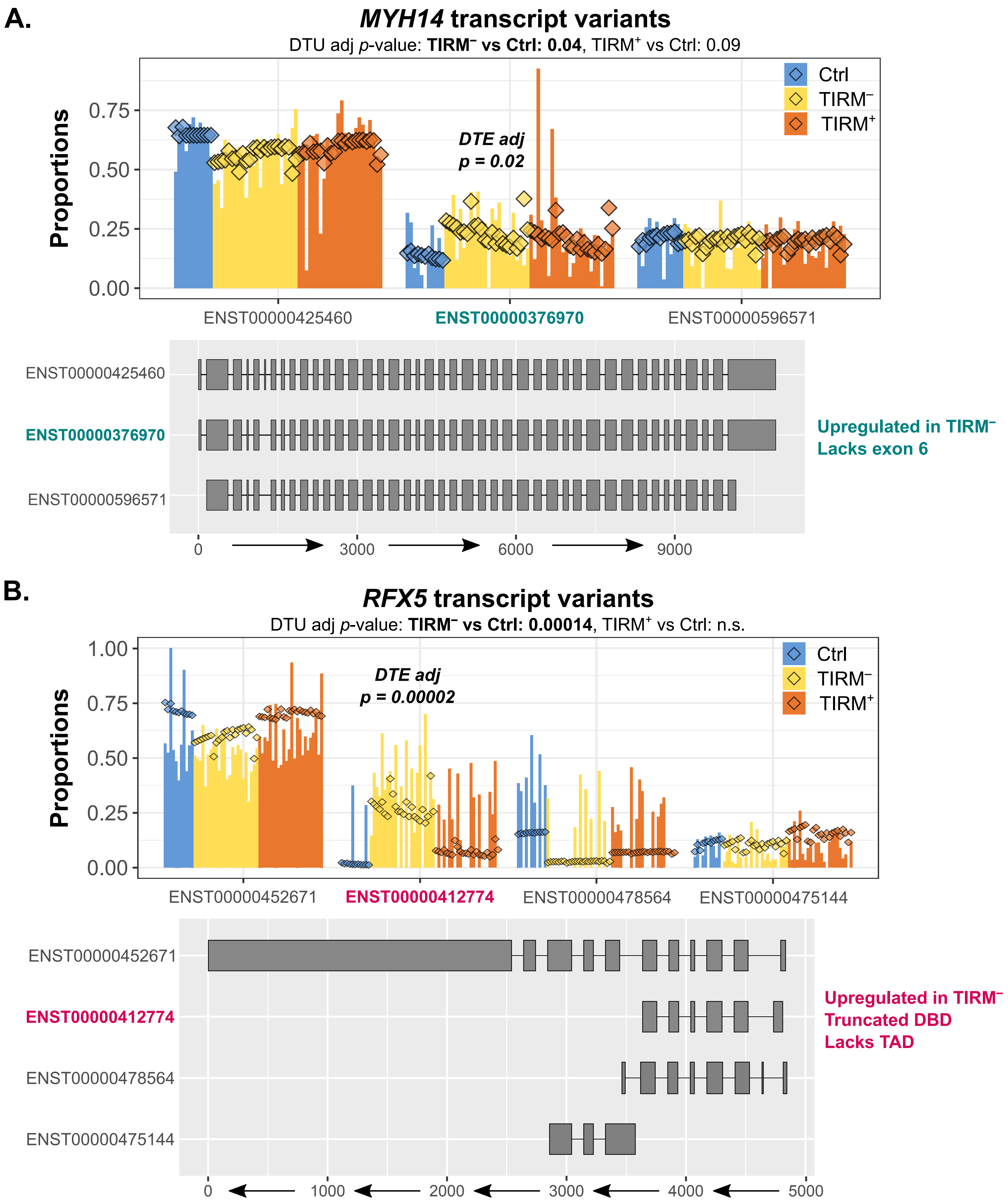

Supplementary Figure 1: DRIMSeq alternative splicing predictions for MYH14 and RFX5.
(A, B) Bar plots generated using our bespoke software (Supplementary File 1) display the proportion of total gene expression for (A) MYH14 and (B) RFX5 accounted for by the major transcript variants, across 11 control samples (blue), 24 TIRM$^-$ FSHD samples (yellow) and 24 TIRM$^+$ FSHD samples (orange). Transcript structures are displayed beneath each plot and coloured text highlights and explains the transcript that is significantly altered in its expression in TIRM$^-$ (yellow) or TIRM$^+$ (orange). At the top of each plot adjusted p-values are displayed denoting the significance of differential transcript usage (DTU) for the gene between control and TIRM$^-$ and control and TIRM$^+$ samples (adjusted for age, sex and multiple comparisons). On each plot is displayed the adjusted p-value for differential transcript expression (DTE) of the highlighted transcript in the comparison for which DTU is significant, adjusting for age, sex and multiple comparisons. DBD = DNA-binding domain; TAD = transactivation domain.
