# Supplementary material for: FSHD muscle shows perturbation in fibroadipogenic progenitor cells, mitochondrial function and alternative splicing independently of inflammation
Source: Hum Mol Genet. 2023 Oct 19;33(2):182–97. doi: 10.1093/hmg/ddad175 (PMC10772042; doi:10.1093/hmg/ddad175)
Supplement: Engquist_et_al_ddad175_2023_Instructions_for_using_Supplementary_Files_S1_and_S2_ddad175 [file engquist_et_al_ddad175_2023_instructions_for_using_supplementary_files_s1_and_s2_ddad175.docx]

**Instructions for using Supplementary Files S1 and S2**

**Supplementary File S1:** *Data visualisation software for alternative splicing in FSHD and control muscle biopsies.*

There is a .Rd data file containing the results of alternative splicing analysis in the muscle biopsy dataset (DTE_DTU_shiny_files.rd) and a .R file written using the *shiny* package in R (shiny_DTE_DTU.r). Running the .R file after loading the .Rd file into an R terminal will launch a graphical user interface, allowing navigation of the results of our alternative splicing analysis, displaying for each gene with evidence of DTU: relative proportion of transcripts, a map of the transcript structure and 2 tables displaying DTU/DTE results for comparisons of TIRM^–^ versus control and TIRM^+^ versus control separately.

**Supplementary File S2:** *Data visualisation software for alternative splicing in FSHD and control PBMCs.*

There is a .Rd data file containing the results of alternative splicing analysis in the PBMC dataset (DTE_DTU_shiny_files_PBMCs.Rd) and a .R file written using the *shiny* package in R (shiny_DTE_DTU_PBMC.r). Running the .R file after loading the .Rd file into an R terminal will launch a graphical user interface, allowing navigation of the results of our alternative splicing analysis, displaying for each gene with evidence of DTU: relative proportion of transcripts, a map of the transcript structure and a table displaying DTU/DTE results for comparison of FSHD versus control PBMCs.
